# Noncoding RNAs-mediated overexpression of KIF14 is associated with tumor immune infiltration and unfavorable prognosis in lung adenocarcinoma

**DOI:** 10.18632/aging.204332

**Published:** 2022-10-11

**Authors:** Hong Wang, Fangting Tang, Ping Tang, Liang Zhang, Qixin Gan, Yuejun Li

**Affiliations:** 1 Department of Oncology, The Third Affiliated Hospital of Hunan University of Chinese Medicine, Zhuzhou, Hunan Province 412000, PR China; 2 Department of Oncology, The First Affiliated Hospital of Hunan College of Traditional Chinese Medicine, Zhuzhou, Hunan Province 412000, PR China; 3 Department of Radiology, The Third Affiliated Hospital of Hunan University of Chinese Medicine, Zhuzhou, Hunan Province 412000, PR China; 4 Department of Radiology, The First Affiliated Hospital of Hunan College of Traditional Chinese Medicine, Zhuzhou, Hunan Province 412000, PR China

**Keywords:** KIF14, lung adenocarcinoma, noncoding RNAs, hsa-miR-101-3p

## Abstract

Kinesin family member 14 (KIF14) is potentially oncogenic and acts as a chromokinesin via binding to microtubules and chromatin during the bipolar spindle formation. KIF14 overexpression is a significant prognostic biomarker in various cancers. However, the expression, prognosis, mechanism, and tumor immune regulation of KIF14 in lung adenocarcinoma (LUAD) remain obscure. Our results demonstrated that KIF14 was upregulated in a variety of cancers, including LUAD. High-expression of KIF14 in LUAD was associated with pathological tumor stage, N stage and unfavorable prognosis. Both univariate and multivariate Cox regression results demonstrated that KIF14 was a significant independent risk factor influencing the prognosis of LUAD patients. The most promising upstream ncRNA-associated pathway of KIF14 in LUAD was determined to be GSEC/TYMSOS-hsa-miR-101-3p axis according to the starBase and The Cancer Genome Atlas databases. Furthermore, upregulation of KIF14 in LUAD was positively correlated with tumor mutation burden, microsatellite instability, immune checkpoint-related gene expression, immune cell biomarkers, and tumor immune cell infiltration. This study reveals that ncRNAs-mediated overexpression of KIF14 is associated with tumor immune infiltration and unfavorable prognosis in LUAD.

## INTRODUCTION

Lung cancer is a frequently diagnosed cancer worldwide, and the leading cause of cancer-related death [1]. Lung adenocarcinoma (LUAD), which comprises 40% of all lung cancers cases, is responsible for over 500,000 deaths per year worldwide [2]. Considerable efforts have been made to develop novel anticancer drugs for LUAD patients over the last decade, but their 5-year overall survival (OS) rates are still poor, probably due to the high heterogeneity of the disease and lack of effective diagnostic and prognostic biomarkers [3–6]. The advancement of immunotherapy can serve as a promising rescue option for LUAD patients [3]. Several studies have demonstrated that immune checkpoint inhibitors could improve OS, progression-free survival (PFS), and objective response rates in patients with non-small cell lung cancer when used in the first- or subsequent-line settings [7–9]. However, a substantial portion of patients failed to respond to this therapy [5, 7–10]. Therefore, it is of utmost urgency to develop novel therapeutic targets and reliable biomarkers for LUAD treatment.

Kinesin family member 14 (KIF14), is an emerging molecular motor that involved in cytokinesis completion, midbody formation, chromosome segregation, mitotic spindle formation and cargo-containing vesicle transport [11–14]. KIF14 is potentially oncogenic and can act as a chromokinesin via binding to microtubules and chromatin during the bipolar spindle formation [11, 14, 15]. High expression of KIF14 is demonstrated to be a significant prognostic biomarker in various cancers, including pancreatic adenocarcinoma (PAAD), hepatocellular carcinoma, cervical cancer and ovarian cancer [16–19]. Knocking-down of KIF14 could result in the failure of cytokinesis and inhibition of cancer cell growth, while overexpression of KIF14 induced cancer cell proliferation [15, 20]. As an ATPase, it also has potential as a therapeutic target [21]. However, the expression, prognosis, mechanisms and tumor immune regulation of KIF14 in patients with LUAD remain obscure.

In the present study, both expression and survival analyses were conducted on KIF14 in pan-cancer with an emphasis on LUAD. Moreover, the association between KIF14 and long non-coding RNAs (lncRNA) or microRNAs (miRNAs) in LUAD were evaluated. In addition, we explored the relationships of KIF14 with immune checkpoints-related genes, immune cell biomarkers and immune cell infiltration (ICI) in LUAD. The results indicated that lncRNAs- and miRNAs-regulated overexpression of KIF14 was associated with tumor immune infiltration and unfavorable prognosis in patients with LUAD.

## RESULTS

### The expression of KIF14 in pan-cancer

**Figure 1:**
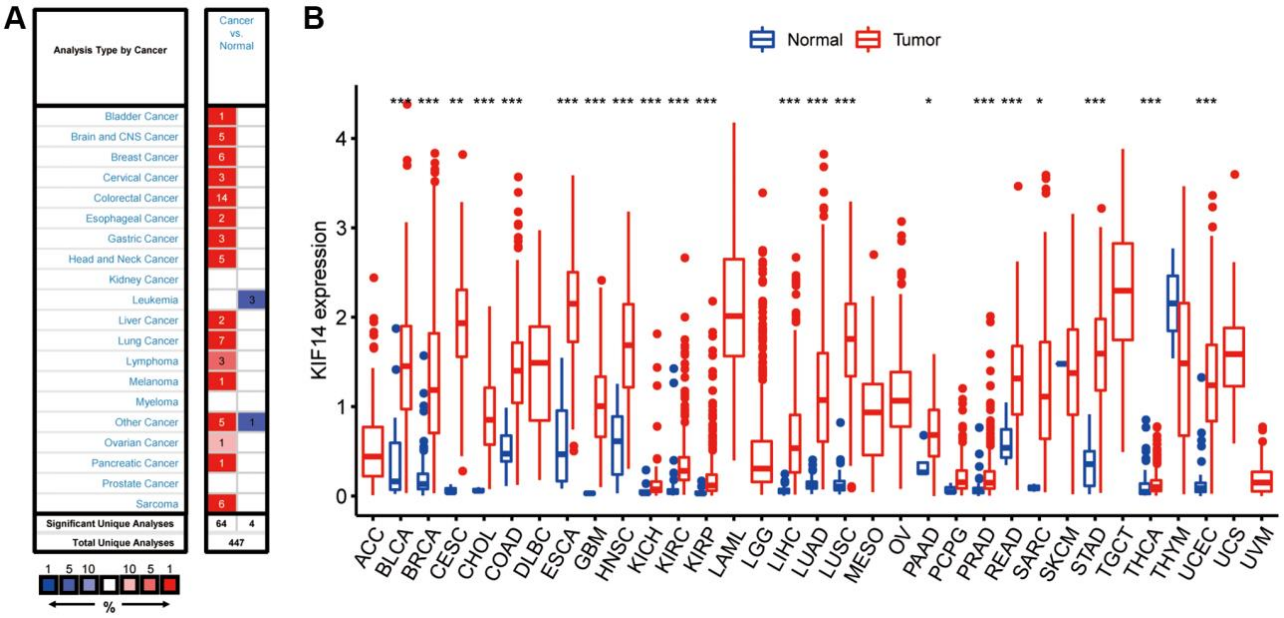
**Expression of KIF14 in pan-cancers.** (**A**) Upregulated or downregulated levels of KIF14 in different tumors collected from the Oncomine database. (**B**). Expression levels of KIF14 in various tumors from The Cancer Genome Atlas (TCGA) database analyzed by R package. ^*^P-value < 0.05, ^**^P-value < 0.01, ^***^P-value < 0.001.

Expression analysis of KIF14 through the Oncomine database (http://www.oncomine.org) revealed that KIF14 was overexpressed in numerous cancers, including LUAD, compared with their respective adjacent tissues (Figure 1A). The results of difference expression analyses through The Cancer Genome Atlas (TCGA) database (http://portal.gdc.cancer.gov) confirmed that KIF14 was upregulated not only in LUAD, but also in uterine corpus endometrial carcinoma (UCEC), thyroid carcinoma (THCA), stomach adenocarcinoma (STAD), sarcoma (SARC), rectum adenoma (READ), prostate adenocarcinoma (PRAD), PAAD, lung squamous cell carcinoma (LUSC), liver hepatocellular carcinoma (LIHC), kidney renal papillary cell carcinoma (KIRP), kidney renal clear cell carcinoma (KIRC), head and neck squamous cell carcinoma (HNSC), glioblastoma multiforme (GBM), esophageal carcinoma (ESCA), colon adenocarcinoma (COAD), cholangiocarcinoma (CHOL), endocervical adenocarcinoma (CESC), breast invasive carcinoma (BRCA), cervical squamous cell carcinoma and bladder urothelial carcinoma (BLCA) (Figure 1B).

### High-expression of KIF14 predicts unfavorable prognosis

**Figure 2:**
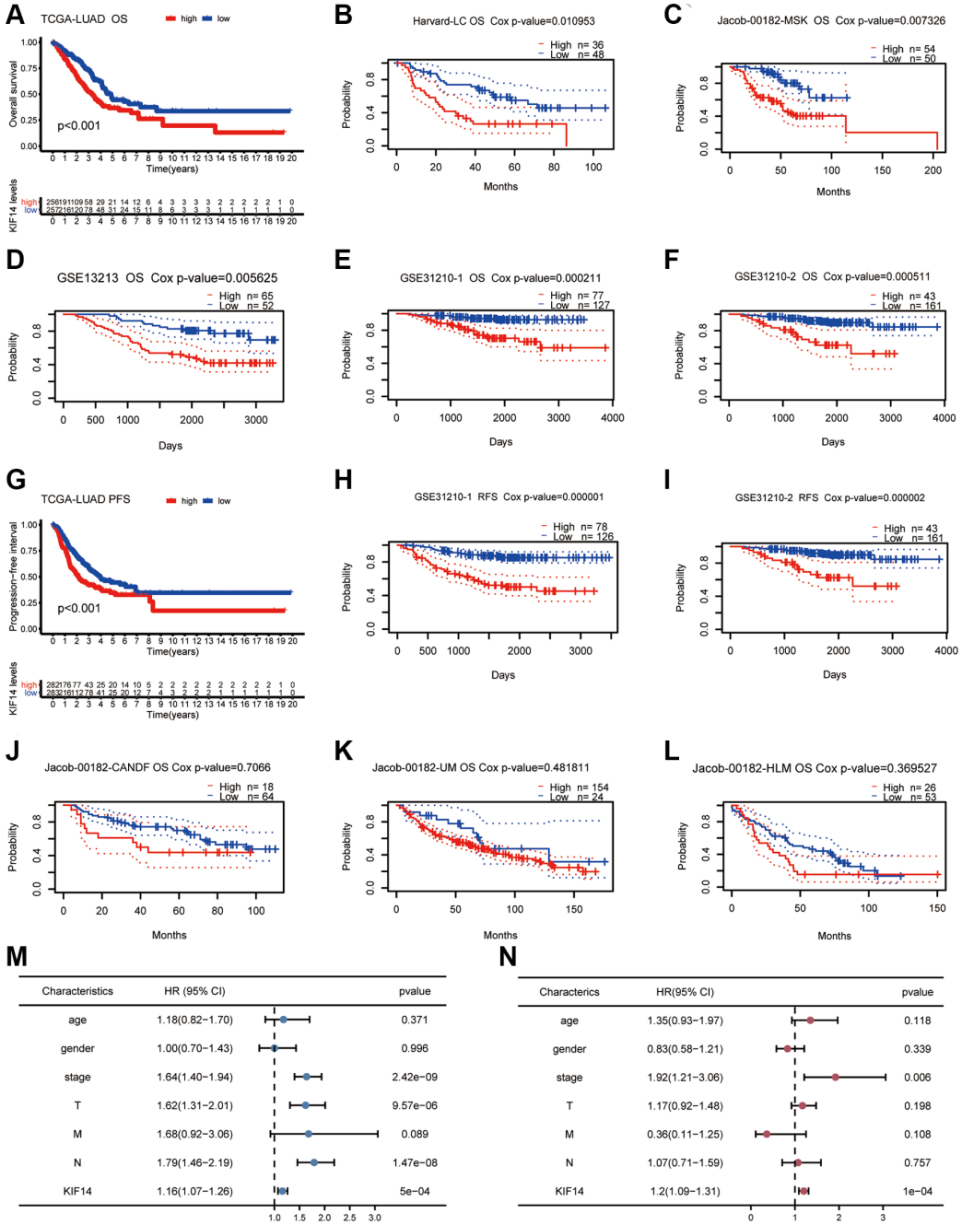
**Survival analyses of KIF14 in LUAD.** (**A**–**F**) K-M plots for OS in different LUAD cohorts (TCGA-LUAD cohort, Harvard -LC cohort, jacob-00182-MSK cohort, GSE13212 cohort, GSE31210-1 cohort, and GSE31210-2 cohort). (**G**–**I**) Survival curves of PFS, RFS in LUAD cohorts (TCGA-LUAD cohort, GSE31210-1 cohort, and GSE31210-2 cohort). (**J**–**L**) OS survival analyses of KIF14 in LUAD cohorts with negative results (jacob-00182-CANDF cohort, jacob-00182-UM cohort and jacob-00182-HLM cohort). (**M**, **N**) Univariate (**M**) and multivariate (**N**) Cox regression analyses to evaluate the independent factors of KIF14 in TCGA-LUAD cohort.

The Kaplan-Meier (K-M) survival analysis was conducted to assess the correlation between KIF14 expression and survival outcomes (PFS and OS) in pan-cancer according to the TCGA database. Both OS and PFS results demonstrated that high KIF14 expression could predict unfavorable prognosis in LUAD, PAAD, mesothelioma (MESO), LIHC, KIRP, KIRC, and adrenocortical carcinoma (ACC) (Figure 2A and 2G, Supplementary Figures 1 and 2). Next, we verified the results of LUAD via the PrognoScan database (http://dna00.bio.kyutech.ac.jp/PrognoScan/index.html) [22] (Table 1). Notably, high KIF14 expression was significantly correlated with shorter OS (Figure 2B–2F) and relapse-free survival (RFS) (Figure 2H and 2I) in seven out of ten independent cohorts. In the remaining three cohorts [23] (jacob-00182-CANDF, jacob-00182-UM, and jacob-00182-HLM cohorts, Figure 2J–2L), the OS of KIF14 upregulation group was still shorter than that of KIF14 downregulation group, but no significant difference was observed. For TCGA-LUAD cohort, both univariate and multivariate Cox regression analysis revealed that KIF14 was an independent prognostic factor for OS (Figure 2M and 2N), with Akaike information criterion (AIC) = 1094.23, *p*-value = 1e-04. Altogether, these results demonstrate that KIF14 can serve as a prognostic biomarker for patients with LUAD.

**Table 1:** Expression of KIF14 in lung adenocarcinoma in PrognoScan database.

**Cancer**	**Dataset**	**Endpoint**	**Probe ID**	***N***	**Cox *P*-value**
LUAD	jacob-00182-CANDF	OS	206364_at	82	0.7066
LUAD	HARVARD-LC	OS	34563_at	84	0.003813
LUAD	jacob-00182-HLM	OS	206364_at	79	0.369527
LUAD	jacob-00182-MSK	OS	206364_at	104	0.001179
LUAD	GSE13213	OS	A_23_P149668	117	0.001809
LUAD	GSE31210	OS	236641_at	204	0.000691
LUAD	GSE31210	OS	206364_at	204	0.001082
LUAD	GSE31210	RFS	206364_at	204	0.000028
LUAD	GSE31210	RFS	236641_at	204	0.000005
LUAD	jacob-00182-UM	OS	206364_at	178	0.481811

Abbreviations: LUAD: Lung adenocarcinoma; OS: Overall survival; RFS: Relapse free survival.

### Overexpression of KIF14 correlated with adverse clinical characteristics in LUAD

**Figure 3:**
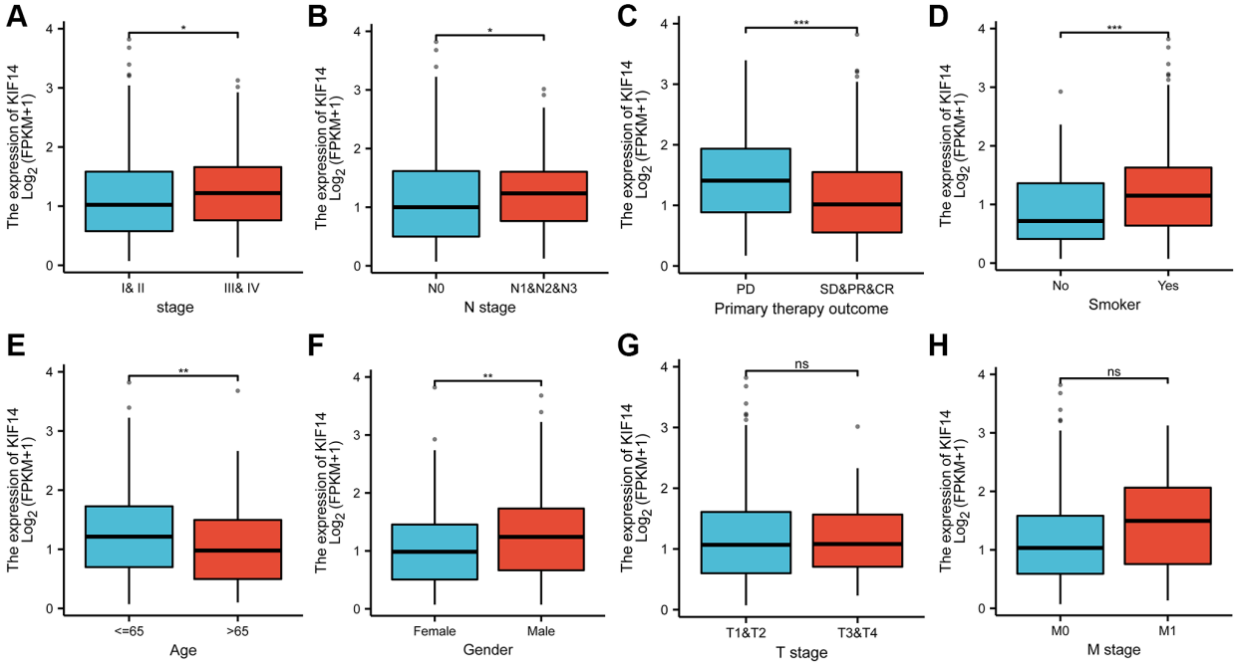
**The correlations between KIF14 expression and pathological characteristics in LUAD.** Upregulation of KIF14 is positively related to higher pathological stage (**A**), *N* stage (**B**), uncontrolled disease (**C**), smoking (**D**), younger patients(**E**), and male (**F**) in TCGA-LUAD cohort. The associations between the expression of KIF14 and T stage (**G**) and M stage (**H**) were not statistically significant. ^*^P-value < 0.05, ^**^P-value < 0.01, ^***^P-value < 0.001. Abbreviation: ns: non-statical significant.

The correlation between KIF14 expression and clinical characteristics in LUAD were analyzed. The results showed that KIF14 was positively related to advanced tumor stage (Figure 3A, *p* = 0.021) and lymph node metastasis (Figure 3B, *p* = 0.014). Moreover, high expression of KIF14 were more common in patients with uncontrolled disease (Figure 3C, *p* < 0.001), smoker (Figure 3D, *p* = 0.001), younger patients (Figure 3E, *p* = 0.001), and male (Figure 3F, *p* = 0.001). The associations between the expression of KIF14 and T stage and M stage were not statistically significant (Figure 3G and 3H, *p >* 0.05). Hence, all the above findings suggest that KIF14 might be involved in the tumorigenesis of LUAD.

### Prediction and construction of the ceRNA network of KIF14 in LUAD

#### Identification of upstream miRNAs of KIF14

**Figure 4:**
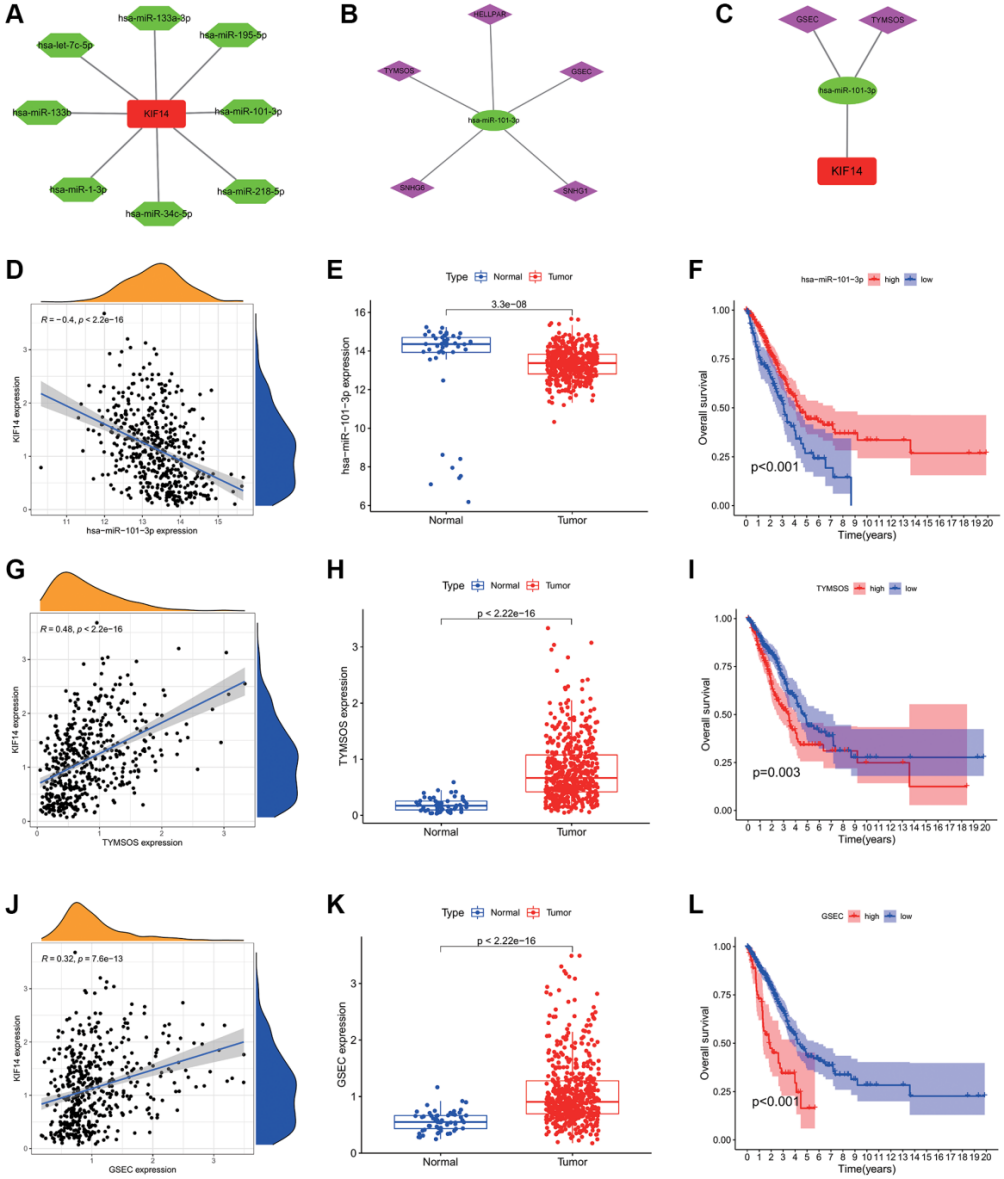
**Construction of the ceRNA network of KIF14 in LUAD.** (**A**) The potential miRNAs-KIF14 regulatory network constructed via Cytoscape tool. (**B**) The potential lncRNAs-hsa-miR-101-3p regulatory network constructed via Cytoscape tool. (**C**) The ceRNA network of GSEC/TYMSOS-hsa-miR-101-3p-KIF14 in LUAD. (**D**) The correlation between hsa-miR-101-3p and KIF14 expression in LUAD. (**E**) Expression levels of hsa-miR-101-3p in LUAD and control tissues in TCGA-LUAD cohort. (**F**) Survival analysis of hsa-miR-101-3p in TCGA-LUAD cohort. (**G**) The correlation between TYMSOS and KIF14 expression in LUAD. (**H**) Expression levels of TYMSOS in LUAD and control tissues in TCGA-LUAD cohort. (**I**) Survival analysis of TYMSOS in TCGA-LUAD cohort. (**J**) The correlation analysis of GSEC and KIF14 expressions in LUAD. (**K**) The differential expression of GSEC in LUAD and normal tissues in TCGA-LUAD cohort. (**L**) Survival analysis of GSEC in TCGA-LUAD cohort.

To explore the upstream miRNAs regulating KIF14 expression, the binding of miRNAs to KIF14 was assessed using the starBase database (http://starbase.sysu.edu.cn) [24], and then visualized in a miRNAs-KIF14 regulatory network constructed by Cytoscape tool [25]. In view of the action mechanisms by which miRNAs negatively regulate KIF14 expression, the miRNAs were then filtered according to the following criteria: (i) downregulation patterns in LUAD, (ii) positive correlation with LUAD patients’ prognosis, and (iii) negative correlation with KIF14 expression. Finally, a total of 7 miRNAs were included (Table 2). Among them, hsa-miR-101-3p was further selected for subsequent analysis, which exhibited the highest correlation with KIF14 expression in LUAD patients (Table 2, Figure 4A and 4D–4F). The binding target sequence information can be found in Supplementary Figure 3.

**Table 2:** Potential upstream miRNAs of KIF14.

**Gene**	**miRNA**	***R*-value**	***P*-value**
KIF14	hsa-miR-101-3p	−0.404705546	0
KIF14	hsa-let-7c-5p	−0.362901213	3.12E-16
KIF14	hsa-miR-133a-3p	−0.352791372	2.28E-15
KIF14	hsa-miR-195-5p	−0.35086189	4.22E-15
KIF14	hsa-miR-218-5p	−0.262940449	6.79E-09
KIF14	hsa-miR-34c-5p	−0.243877863	8.08E-08
KIF14	hsa-miR-133b	−0.223939	8.20E-07

#### Prediction and construction of the ceRNA network of KIF14

**Figure 5:**
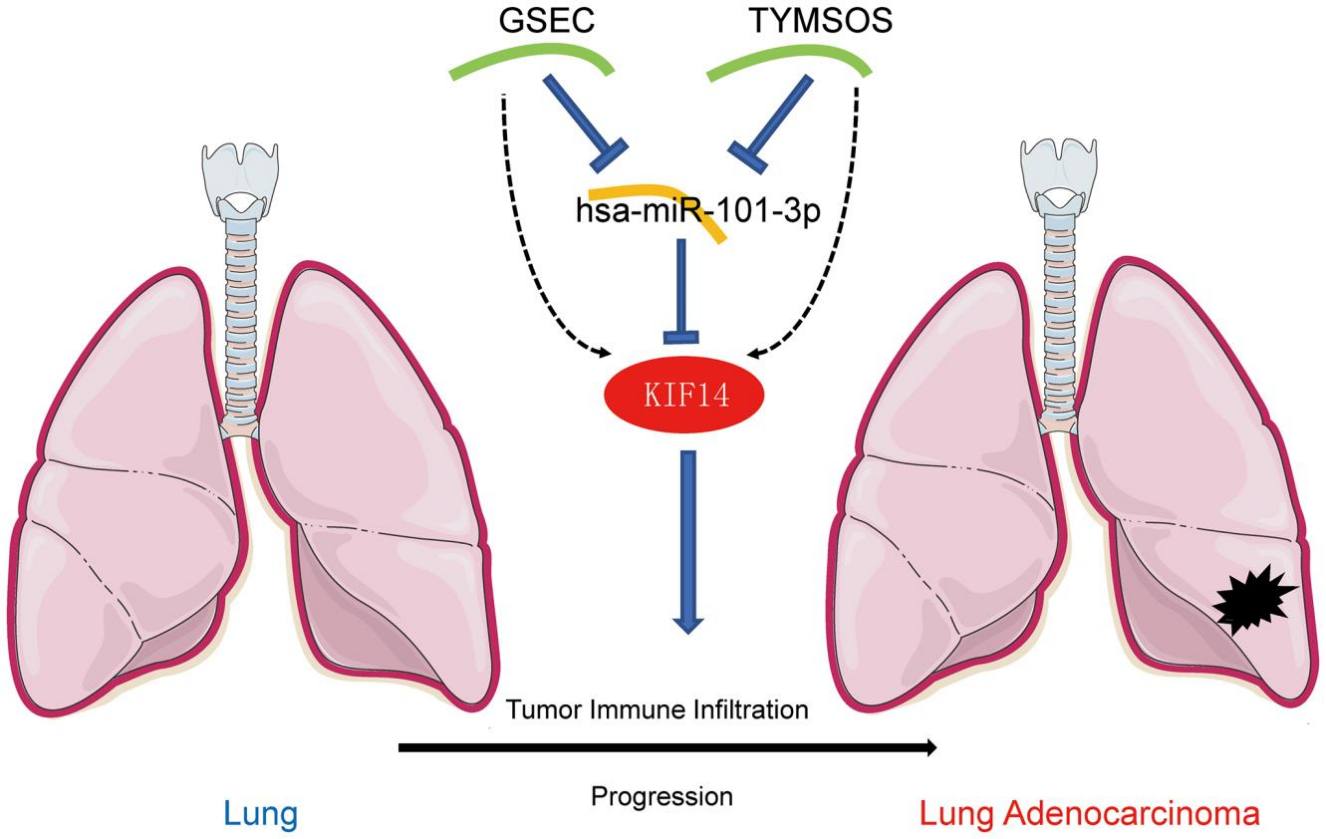
The model of GSEC/TYMSOS-hsa-miR-101-3p axis in tumorigenesis of LUAD.

Through the starBase database, the potential upstream lncRNAs of hsa-miR-101-3p were predicted (Figure 4B). According to the ceRNA hypothesis, these lncRNAs were also filtered by the following criteria [26, 27]: (i) upregulation patterns in LUAD, (ii) positive correlation with KIF14 expression, (iii) negative correlation with hsa-miR-101-3p expression, (iv) negative correlation with LUAD patients’ prognosis, and (v) significant association with unfavorable prognosis in LUAD patients. According to the results listed in Tables 3–5, GSEC and TYMSOS (Figure 4B, 4C and 4G–4L), which were negatively correlated LUAD patients’ OS, were considered as the two most potential upstream lncRNAs of hsa-miR-101-3p/KIF14 axis in LUAD patients (Figure 5), while SNHG6, HELLPAR, and SNHG1 were excluded (Supplementary Figure 4).

**Table 3:** Association analysis between lncRNA and hsa-miR-101-3p or lncRNA and KIF14 in LUAD determined via starBase database.

**lncRNA**	**miRNA**	***R*-value**	***P*-value**
GSEC	hsa-miR-101-3p	−0.224264048	8.45E-07
HELLPAR	hsa-miR-101-3p	−0.243521462	8.45E-08
SNHG1	hsa-miR-101-3p	−0.222764208	1.00E-06
SNHG6	hsa-miR-101-3p	−0.216861364	1.95E-06
TYMSOS	hsa-miR-101-3p	−0.287171466	2.19E-10

**Table 4:** Association analysis between lncRNA and KIF14 in LUAD determined via starBase database.

**lncRNA**	**Gene**	***R*-value**	***P*-value**
SNHG6	KIF14	0.166466613	0.000273472
TYMSOS	KIF14	0.484935757	0
HELLPAR	KIF14	0.51181722	0
GSEC	KIF14	0.32269557	7.57E-13
SNHG1	KIF14	0.368245931	7.11E-17

**Table 5:** Correlation analysis between lncRNA and hsa-miR101-3p in LUAD.

**lncRNA**	**miRNA**	***R*-value**	***P*-value**
SNHG6	hsa-miR-101-3p	−0.216861364	1.95E-06
TYMSOS	hsa-miR-101-3p	−0.287171466	2.19E-10
HELLPAR	hsa-miR-101-3p	−0.243521462	8.45E-08
GSEC	hsa-miR-101-3p	−0.224264048	8.45E-07
SNHG1	hsa-miR-101-3p	−0.222764208	1.00E-06

### Association of KIF14 expression with tumor ICI in LUAD

#### Association of KIF14 expression with TMB and MSI

**Figure 6:**
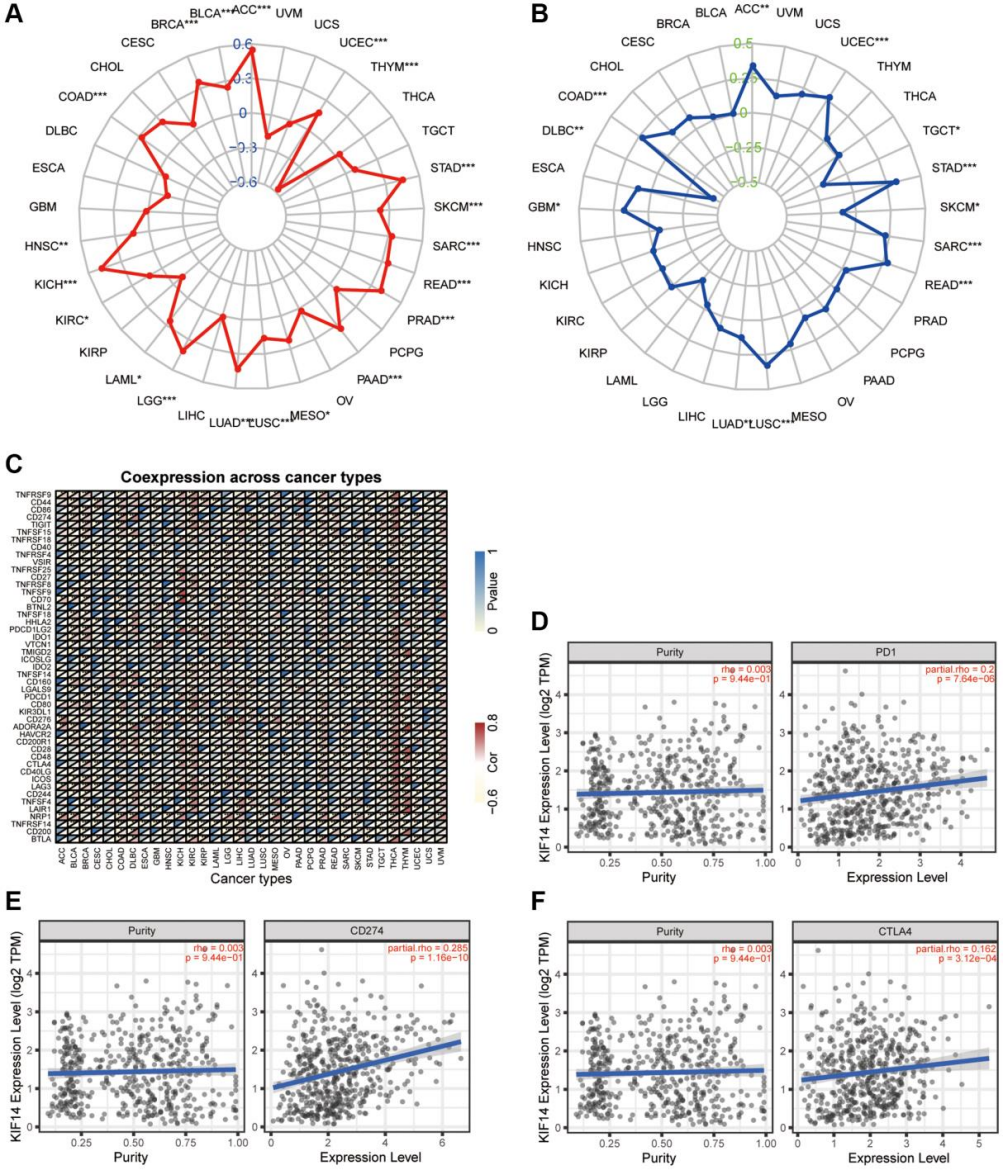
**Association between KIF14 expression and immune infiltration in LUAD.** (**A**–**C**) The association of KIF14 expression with TMB (**A**), MSI (**B**) and immune checkpoint-related genes (**C**) in pan-cancer. (**D**–**F**) The associations of KIF14 expression with PD-1 (**D**), CD274 (**E**) and CTLA4 (**F**) expression in LUAD, which were determined using the TIMER database. ^*^P-value < 0.05, ^**^P-value < 0.01, ^***^P-value < 0.001.

TMB is defined as the distribution density of nonsynonymous mutations in the protein- coding sequences, and is closely association with tumor prognosis. MSI refers to the changes in the length of microsatellite sequences due to deletion or insertion mutations during the process of DNA replication, which is mostly caused by functional defects such as the mismatch repair system (MMRs), and is involved in tumor development. Both TMB and MSI have been found to be closely associated with immunotherapeutic response. The expression of KIF14 was remarkably correlated with TMB in LUAD, UCEC, THYM, STAD, skin cutaneous melanoma (SKCM), SARC, READ, PRAD, PAAD, MESO, LUSC, KIRC, KICH, HNSC, COAD, BRCA, brain lower grade glioma (BLGG), BLCA, acute myeloid leukemia (AML) and ACC (Figure 6A). Meanwhile, the expression of KIF14 was markedly associated with MSI in LUAD, UCEC, testicular germ cell tumors (TGCT), STAD, SKCM, SARC, READ, LUSC, GBM, diffuse large B-cell lymphoma (DLBC) in lymphoid neoplasm, COAD and ACC (Figure 6B). Interestingly, the expression of KIF14 was closely related to both TMB and MSI in LUAD, indicating that KIF14 may be used as a biomarker for immunotherapy in LUAD patients.

#### Relationship between KIF14 and immune checkpoint-related genes in LUAD

Immune checkpoint-related genes, including cytotoxic T-lymphocyte associated protein 4 (CTLA4), programmed cell death-ligand 1 (CD274, PD-L1), and programmed cell death 1 (CD279, PDCD1, PD-1), play vital roles in regulating tumor immune escape [28–30]. Given the tumorigenic role of KIF14 in LUAD, we explored the relationships between these genes and KIF14 in pan-cancer with R. As displayed in Figure 6C, KIF14 expression was significantly correlated with immune checkpoint-related genes in pan-cancer. Importantly, KIF14 was positively correlated with CTLA-4, PD-L1 and PD-1 in LUAD. These relationships were further verified through TIMER database, which was adjusted by tumor purity (Figure 6D–6F). The results suggest that tumor immune escape might responsible for KIF14-mediated tumorigenesis in LUAD patients.

#### KIF14 is positively correlated with tumor ICI in LUAD

To explore the correlation between KIF14 expression and tumor ICI in LUAD, TIMER database was used for the analysis [31]. KIF14 expression was positively related to CD4^+^ T cell, CD8^+^ T cell, neutrophil cell, monocyte cell and NK cell, which negatively correlated to B cell, dendritic cell, and Treg cell (Supplementary Figure 5). The relationships between KIF14 expression and immune cell biomarkers in LUAD were determined by R using the “limma”, “reshape2”, “ggpubr”, and “ggExtra” packages. As demonstrated in Table 6, the expression of KIF14 was positively associated with CD8^+^ T cell (CD8A, CD8B), M1 macrophage (NOS2, PTGS2), and negatively associated with B cell (CD79A, CD19), CD4^+^ T cell (CD4), M2 macrophage (VSIG4, MS4A4A), Neutrophil (CEACAM8, ITGAM, CCR7), dendritic cell (ITGAX, HLA-DPA1, HLA-DRA, HLA-DQB1, HLA-DPB1, CD1C). These findings suggest that the expression of KIF14 can influence tumor ICI in LUAD.

**Table 6:** Association analysis between KIF14 and biomarkers of immune cells in LUAD.

**Immune cell**	**Biomarker**	***R*-value**	***P*-value**
B cell	CD19	−0.073981614	0.090065292
	CD79A	−0.109315105	0.01215151^*a^
CD8+ T cell	CD8A	0.128083306	0.003273869^**a^
	CD8B	0.111475056	0.010542829^*a^
CD4+ T cell	CD4	−0.199707609	4.10E-06^***a^
M1 macrophage	NOS2	0.050735194	0.245319259
	IRF5	−0.022416371	0.607871735
	PTGS2	0.137915682	0.001534231^*a^
M2 macrophage	CD163	0.102762936	0.018434308^*a^
	VSIG4	−0.097925783	0.024740951^*a^
	MS4A4A	−0.074040406	0.089809542
Neutrophil	CEACAM8	−0.355451108	4.14E-17^***a^
	ITGAM	−0.138026586	0.001520742^*a^
	CCR7	−0.202416474	3.01E-06^***a^
Dendritic cell	HLA-DPB1	−0.441527427	0^***a^
	HLA-DQB1	−0.368708739	0^***a^
	HLA-DRA	−0.384973805	0^***a^
	HLA-DPA1	−0.349574843	1.58E-16^***a^
	CD1C	−0.535750371	0^***a^
	NRP1	0.042026124	0.335941429
	ITGAX	−0.007576651	0.862324995

^a^These results are statistically significant. ^*^p-value < 0.05; ^**^p-value < 0.01; ^***^p-value < 0.001.

## DISCUSSION

Increasing evidence have demonstrated KIF14 plays vital roles in regulating tumorigeneses and progression of various cancer [16, 21, 32, 33]. Abnormal expression of KIF14 contributed to unfavorable prognosis in patients with cancer, and was associated with cancer cell proliferation and progression [16–20, 34]. However, the role of KIF14 in mediating LUAD carcinogenesis is still unclarified, and awaits further research.

In the present study, pan-cancer analyses of KIF14 expression were performed via Oncomine and TCGA databases. Survival analyses indicated that high expression of KIF14 was correlated with worse prognosis in various cancer types, including LUAD.

High expression of KIF14 was positively related to advanced tumor stage, N stage, uncontrolled disease, smoking history and male. All the above findings suggest that KIF14 might be involved in the tumorigenesis of LUAD. Moreover, both univariate and multivariate Cox regression analyses demonstrated that KIF14 was an independent prognostic factor for LUAD. Furthermore, survival analyses through the PrognoScan database confirmed that KIF14 contributed to unfavorable prognosis in patients with LUAD. Altogether, these results reveal the oncogenic role of KIF14 in LUAD.

It is well-known that miRNAs participate in tumorigenic process by primarily regulating the abnormal expression of target genes [35–38].To investigate the upstream regulatory mRNAs of KIF14, several prediction programs, such as TargetScan, PicTar, miRanda, microT, miRmap, RNA22 and PITA, were used to assess the binding of miRNAs with KIF14. The identified miRNAs could act as tumor-suppressive molecules in cancer. For instance, hsa-miR-101-3p is involved in LINC00943-mediated gastric cancer cell proliferation and chemosensitivity [39]. Knocking down of hsa-miR-181c-5p depletes pediatric cancer stem-like cells and impairs tumor sphere formation [40]. Taking differential expression, correlation and survival analyses into consideration, hsa-miR-101-3p was identified as the most promising upstream binding tumor suppressive miRNA of KIF14.

Next, based on the ceRNA hypothesis [26], the upstream lncRNAs of hsa-miR-101-3p/KIF14 axis were predicted through the starBase database. Again, taking differential expression, correlation and survival analyses into consideration, GSEC and TYMSOS were identified as the most promising upstream upregulated lncRNAs. Previous studies have shown that GSEC and TYMSOS contribute the proliferation and migration of cancer cells. Matsumura and colleagues reported that GSEC could inhibit the function of DHX36 to promote colon cancer cell migration [41]. Liu and colleagues found that GSEC accelerated the proliferation and invasion of osteosarcoma via regulating miR-588/EIF5A2 axis [42]. TYMSOS was upregulated in gastric cancer cells and functioned as a ceRNA to post-transcriptionally regulate gastric cancer progression [43]. Taken together, GSEC and TYMSOS/hsa-miR-101-3p/KIF14 axis were identified as potential regulatory pathways in LUAD.

Immunotherapy is an emerging novel therapy for various cancers, including LUAD. TMB, MSI, and PD-1/PD-L1 have been proposed as potential biomarkers of immunotherapy in LUAD patients, but the results are still not satisfactory [5]. The discovery of accurate biomarker for immunotherapy remains a great challenge. Our results demonstrated that KIF14 was significantly correlated with TMB, MSI and immune checkpoint-related genes in various cancers. Impressively, KIF14 expression was positively associated with both TMB and MSI as well as CTLA4, PD-L1 and PD-1 in LUAD, which was further verified through the TIMER database. It has been reported that tumor ICI can influence the prognosis of patients with cancer. In this study, both TIMER database and biomarker analyses with R showed that KIF14 expression was positively related to CD8^+^ T cell and negatively associated with dendritic and B cell. These findings indicate that KIF14 expression may influence the tumor ICI of LUAD, thus highlighting its potential as an immunotherapy target.

Although our results improve our understanding of the correlation between KIF14 and LUAD, several limitations should be noted. Firstly, this study was primarily based on the molecular data downloaded from various public databases. Thus, further experimental studies are needed to verify our findings. Secondly, the function and mechanism of how KIF14 promotes the progression of LUAD should also be experimentally investigated.

## CONCLUSIONS

In conclusion, KIF14 was upregulated in various cancers including LUAD, and contributed to unfavorable prognosis in LUAD patients. GSEC and TYMSOS/hsa-miR-101-3p/KIF14 ceRNA network was identified as a novel upstream regulatory mechanism of KIF14 in LUAD. Moreover, KIF14 exerted an oncogenic role via influencing tumor ICI and immune checkpoint-related gene expression. Nevertheless, further experimental studies are warranted to verify our findings in the near future.

## MATERIALS AND METHODS

### Pan-cancer evaluation of KIF14 expression

The transcriptome data, miRNA data of pan-cancer combined with corresponding patients’ records were download from the TCGA database, and then analyzed with “limma” package in R version:3.6.3. *P-*values < 0.05 were deemed as statistically significant. Then, the mRNA levels of KIF14 in pan-cancers were analyzed using the Oncomine database (http://www.oncomine.org), with thresholds of *p*-value = 0.001 and fold change = 1.5.

### Survival analysis of KIF14 in LUAD

The Kaplan-Meier (K-M) survival analysis was used to assess the differences between the high-expression group and low-expression groups based on median expression of KIF14 via the “survminer” and “survival” packages in R. Next, univariate and multivariate Cox regression analyses were conducted to explore the relationships among KIF14 expression, clinical prognostic indicators and survival time in LUAD patients, which was generated via the “survival” package in R with *p*-values < 0.05 regarded as statistical significance. The correlations between KIF14 and survival endpoints in LUAD was then confirmed using the PrognoScan database, with a fixed threshold of Cox *p*-value < 0.05.

### Evaluation of the correlation between KIF14 expression and clinicopathological features

To evaluate the correlation between KIF14 expression level and clinicopathological features (e.g., TNM stage and age in diagnosis), Chi-squared tests were performed via using the “beeswarm” package in R.

### Candidate miRNA prediction

Upstream binding miRNAs of KIF14 in LUAD were assessed using the starBase database (http://starbase.sysu.edu.cn) that involves TargetScan, PicTar, miRanda, microT, miRmap, RNA22 and PITA programs. Only the miRNAs identified from at least 2 of the above-mentioned programs were subjected to further analysis. The correlation analysis of miRNAs with KIF14 was performed according to their negative regulation of a target mRNA and those differentially expressed in tumor tissue than in normal tissues, which was carried out via the “limma”, “reshape2”, “ggpubr”, “ggExtra” packages in R based on TCGA-LUAD dataset. The survival analyses of miRNAs in LUAD were performed using the “survminer” and “survival” packages in R, with statistical significance set as log rank *p*-value < 0.05. Hsa-miR-101-3p showed highest correlation with KIF14, lowest expression in LUAD tissues and most favorable prognosis, which was selected as the candidate miRNA for KIF14.

### Candidate lncRNA prediction and ceRNA network construction

StarBase database was also utilized to identify candidate lncRNAs bound to hsa-miR-101-3p, which included the correlation analysis of lncRNA-KIF14 with lncRNA-hsa-miR-101-3p in LUAD. Then, the correlation, expression and survival analyses of candidate lncRNAs were performed based on TCGA-LUAD dataset. Finally, the competing endogenous RNA (ceRNA) network of KIF14 was established via Cytoscape tool.

### Evaluation of tumor immune infiltration

Tumor mutation burden (TMB) is defined as the total number of somatic mutations per megabase of a cancer genome [44]. Microsatellite instability (MSI) refers to the insertion or deletion of one or more repeat units in tumor cells [45]. Both TMB and MSI have been regarded as potential biomarkers for predicting responses to immunotherapy. The TMB and MSI values of each tumor in pan-caner were calculated, and their association with KIF14 expression were analyzed using the Spearman rank correlation coefficient. The radar charts were generated with “fmsb” package in R.

TIMER (http://timer.cistrome.org/), a website for the comprehensive analysis of ICI, was employed to determine the association between KIF14 expression and immune checkpoint-related gene expression or tumor ICI in LUAD. Level of statistical significance was set as *p*-value < 0.05.

### Statistical analysis

The difference between normal and pan-cancer were calculated with “limma” package in R. For survival analyses, log rank test was employed for comparison. Univariate and multivariate analyses were carried out via Cox logistic regression model to identify independent clinicopathologic characteristics. The Chi-squared test was applied to evaluate the significant association between KIF14 expression and clinicopathological characteristics. The correlation of KIF14 expression with immune-related genes or tumor ICI was determined via the Spearman’s correlation analysis. A *p*-value < 0.05 was regarded as statistical significance.

## Supplementary Materials

Supplementary Figures
