# Supplementary material for: Noncoding RNAs-mediated overexpression of KIF14 is associated with tumor immune infiltration and unfavorable prognosis in lung adenocarcinoma
Source: Aging (Albany NY). 2022 Oct 11;14(19):8013–31. doi: 10.18632/aging.204332 (PMC9596199; doi:10.18632/aging.204332)
Supplement: Supplementary Figures [file aging-14-204332-s001.pdf]

SUPPLEMENTARY FIGURES

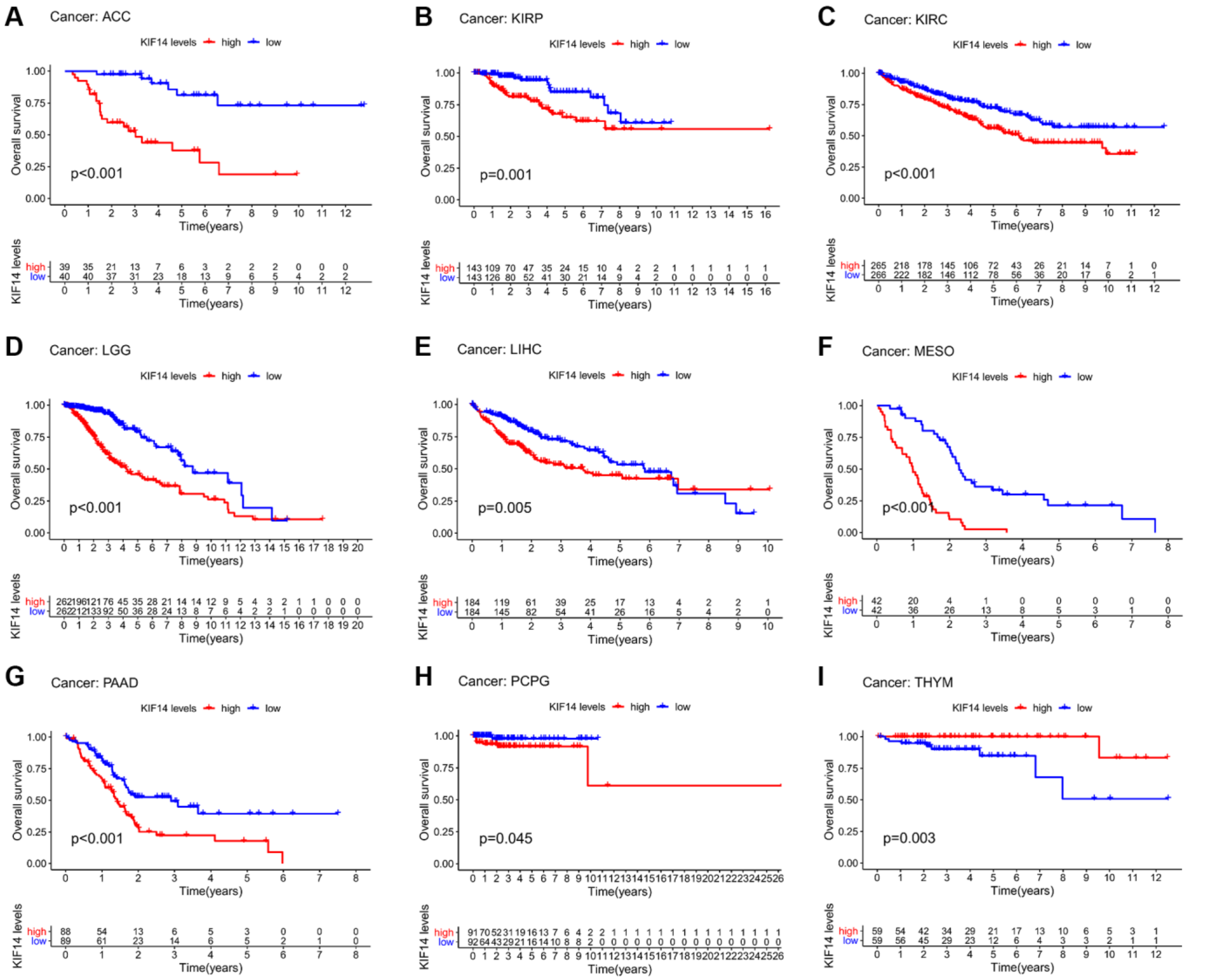

Supplementary Figure 1. Overall survival analyses of KIF14 in pan-cancer. K-M method was used for constructing survival curves. (**A**) Adrenocortical carcinoma (ACC), (**B**) Kidney renal papillary cell carcinoma (KIRP), (**C**) Kidney renal clear cell carcinoma (KIRC), (**D**) Brain Lower Grade Glioma (LGG), (**E**) Liver hepatocellular carcinoma (LIHC), (**F**) Mesothelioma (MESO), (**G**) Pancreatic adenocarcinoma (PAAD), (**H**) Pheochromocytoma and Paraganglioma (PCPG), (**I**) Thymoma (THYM).

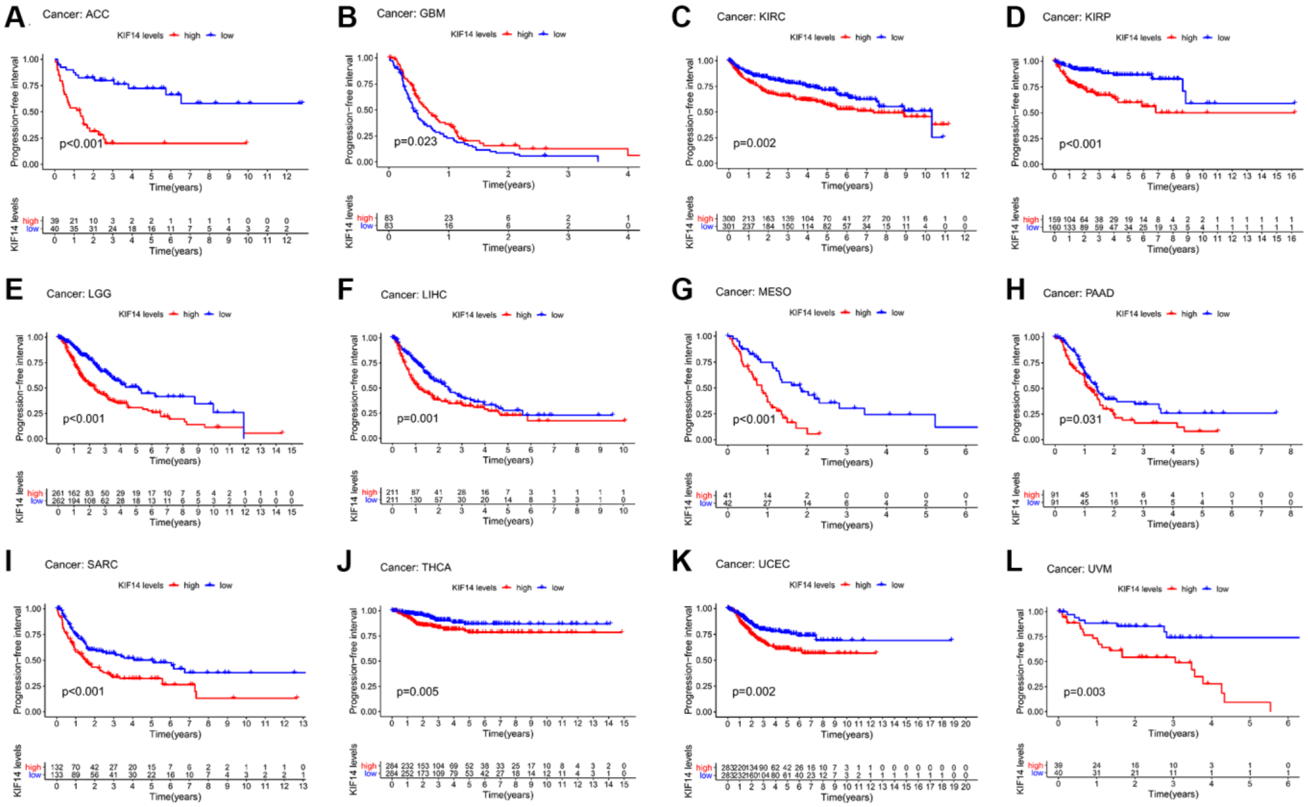

Supplementary Figure 2. Progression-free survival analyses of KIF14 in pan-cancer. K-M method was used for constructing survival curves. (**A**) Adrenocortical carcinoma (ACC), (**B**) Glioblastoma multiforme (GBM), (**C**) Kidney renal clear cell carcinoma (KIRC), (**D**) Kidney renal papillary cell carcinoma (KIRP), (**E**) Brain Lower Grade Glioma (LGG), (**F**) Liver hepatocellular carcinoma (LIHC), (**G**) Mesothelioma (MESO), (**H**) Pancreatic adenocarcinoma (PAAD), (**I**) Sarcoma (SARC), (**J**) Thyroid carcinoma (THCA), (**K**) Uterine Corpus Endometrial Carcinoma (UCEC), (**L**) Uveal Melanoma (UVM).

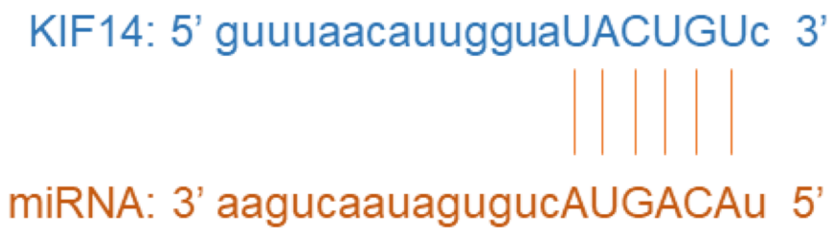

Supplementary Figure 3. The binding target sequence between has-miR-101-3p and 3'UTR of KIF14.

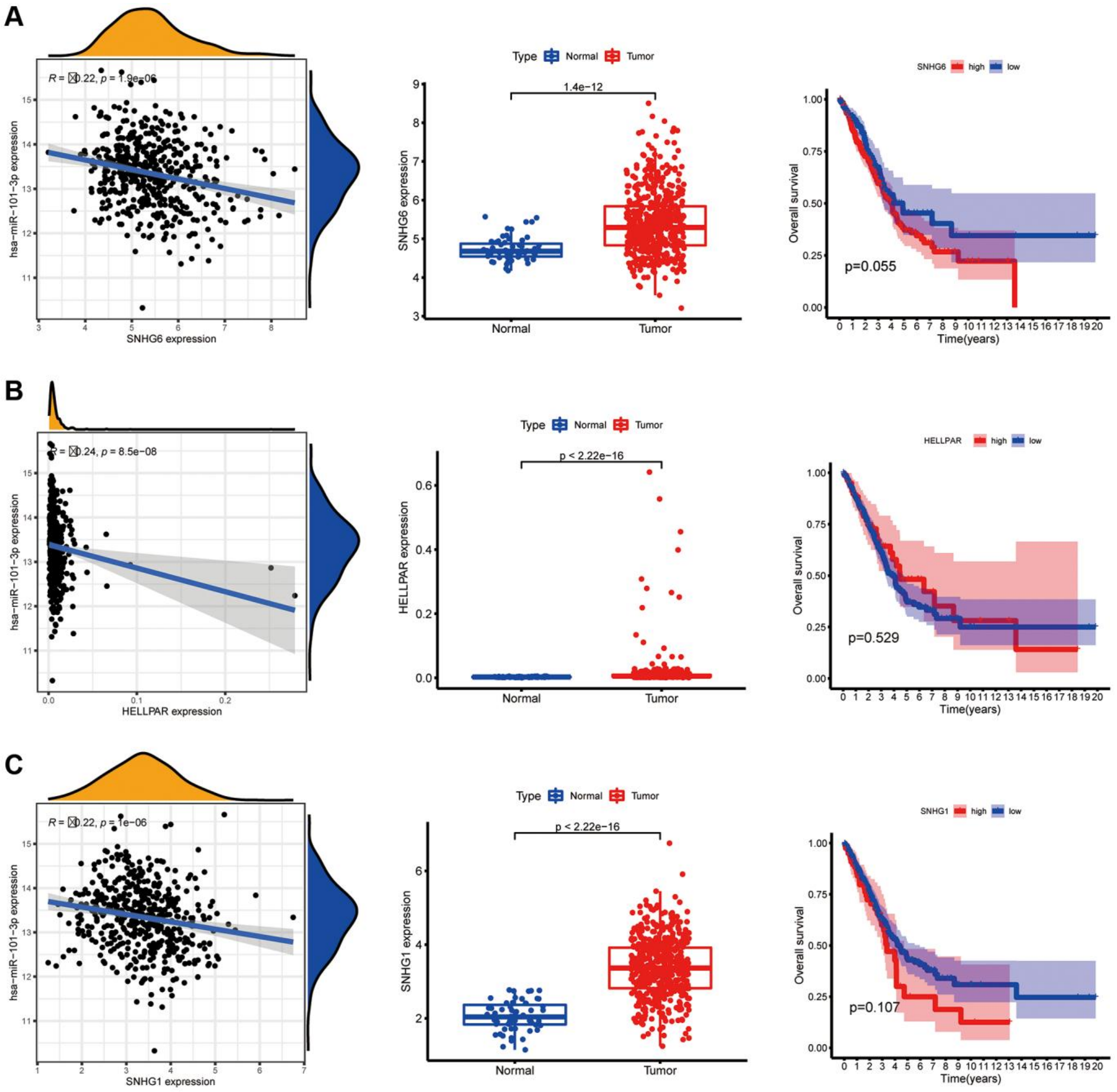

Supplementary Figure 4. The correlation, expression and survival analysis of predicted lncRNAs in TCGA-LUAD cohort. (**A**) The correlation between SNHG6 and KIF14 expression, differential expression and survival analysis of SNHG6 in TCGA-LUAD cohort. (**B**) The correlation between HELLPAR and KIF14 expression, differential expression and survival analysis of SNHG6 in TCGA-LUAD cohort. (**C**) The correlation between SNHG1 and KIF14 expression, differential expression and survival analysis of SNHG6 in TCGA-LUAD cohort.

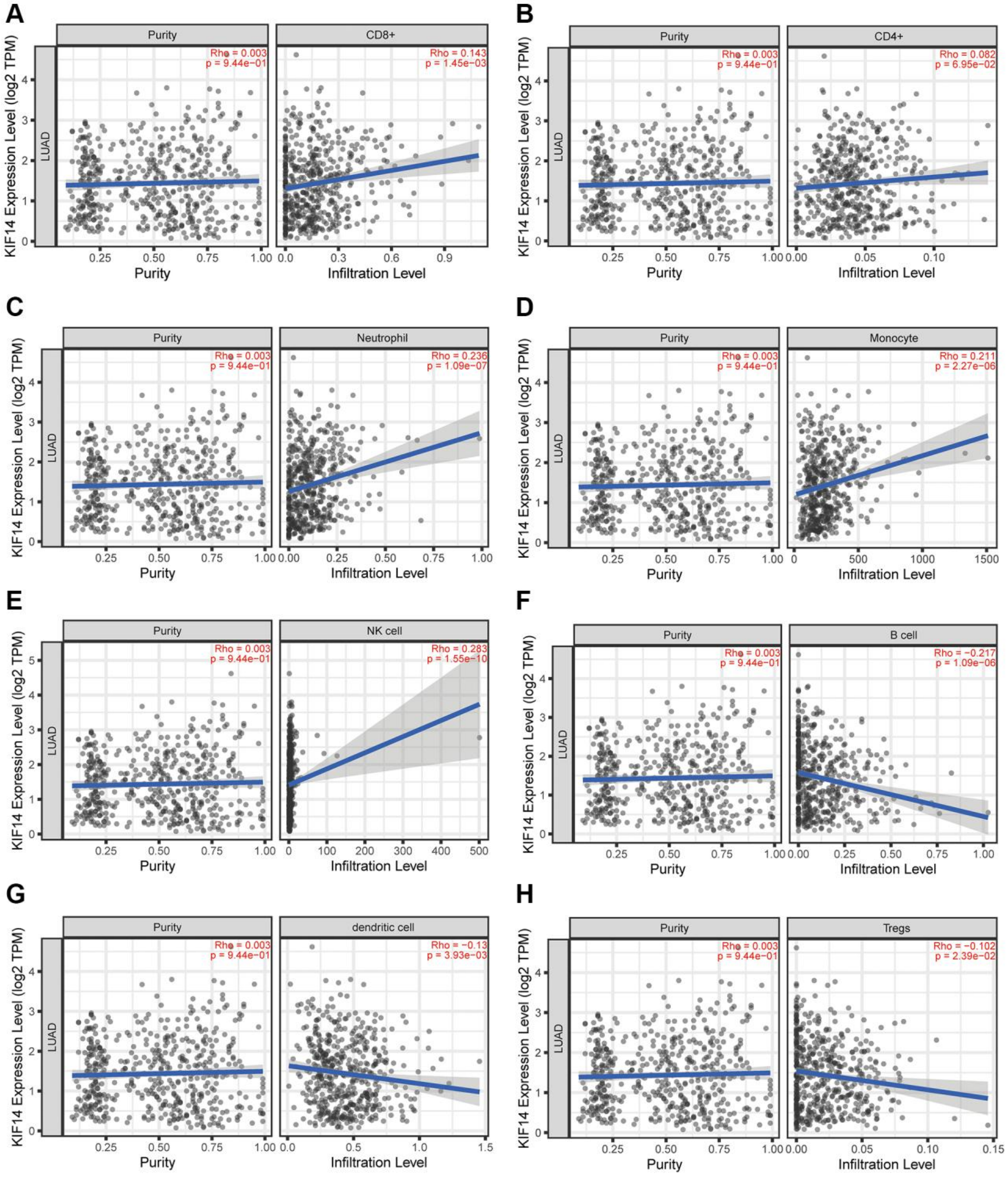

Supplementary Figure 5. Correlation between KIF14 expression and ICI in LUAD. KIF expression was positively correlated with CD8+ T cell (**A**), CD4+ T cell (**B**), neutrophil cell (**C**), monocyte cell (**D**) and NK cell (**E**), while negatively correlated with B cell (**F**), dendritic cell (**G**), and Treg cell (**H**) infiltration in LUAD, which were determined using the TIMER database.
